# Improved quantitative parameter estimation for prostate *T*_2_ relaxometry using convolutional neural networks

**DOI:** 10.1007/s10334-024-01186-3

**Published:** 2024-07-23

**Authors:** Patrick J. Bolan, Sara L. Saunders, Kendrick Kay, Mitchell Gross, Mehmet Akcakaya, Gregory J. Metzger

**Affiliations:** 1 Center for Magnetic Resonance Research, University of Minnesota, 2021 6th Street SE, Minneapolis, MN 55455 USA; 2 Department of Radiology, University of Minnesota, Minneapolis, MN USA; 3 Department of Biomedical Engineering, University of Minnesota, Minneapolis, MN USA; 4 Department of Electrical and Computer Engineering, University of Minnesota, Minneapolis, MN USA

**Keywords:** Magnetic resonance imaging, Prostate, Relaxometry, Neural networks, *T*_2_ mapping

## Abstract

**Objective:**

Quantitative parameter mapping conventionally relies on curve fitting techniques to estimate parameters from magnetic resonance image series. This study compares conventional curve fitting techniques to methods using neural networks (NN) for measuring *T*_2_ in the prostate.

**Materials and methods:**

Large physics-based synthetic datasets simulating *T*_2_ mapping acquisitions were generated for training NNs and for quantitative performance comparisons. Four combinations of different NN architectures and training corpora were implemented and compared with four different curve fitting strategies. All methods were compared quantitatively using synthetic data with known ground truth, and further compared on in vivo test data, with and without noise augmentation, to evaluate feasibility and noise robustness.

**Results:**

In the evaluation on synthetic data, a convolutional neural network (CNN), trained in a supervised fashion using synthetic data generated from naturalistic images, showed the highest overall accuracy and precision amongst the methods. On in vivo data, this best performing method produced low-noise *T*_2_ maps and showed the least deterioration with increasing input noise levels.

**Discussion:**

This study showed that a CNN, trained with synthetic data in a supervised manner, may provide superior *T*_2_ estimation performance compared to conventional curve fitting, especially in low signal-to-noise regions.

**Supplementary Information:**

The online version contains supplementary material available at 10.1007/s10334-024-01186-3.

## Introduction

Quantitative *T*_2_ mapping provides a more objective and potentially sensitive imaging biomarker for diagnosis and grading of prostate cancer compared to qualitative *T*_2_-weighted imaging [1–5]. Mapping is conventionally performed in a two-step process. The first step generates a series of images with increasing echo times, typically using a multi-echo spin-echo acquisition. The second step performs curve fitting on a pixel-by-pixel basis across the image series to estimate the exponential signal decay constant, *T*_2_. This two-step processing is common to a larger group of quantitative MR methods, including other relaxometry applications (*T*_1_, *T*_2_, *T*_2_^*^, *T*_1rho_, etc.), estimation of apparent diffusion coefficient or intravoxel incoherent motion (IVIM) parameters from diffusion-weighted images (DWI), and measurement of flip angle maps for system calibrations. The fitting step requires images with a high signal-to-noise ratio (SNR) and a wide range of echo times to accurately estimate *T*_2_ values. This need for multiple images and high SNR leads to long acquisition times, which limits the adoption of these potentially valuable measurements in both clinical and research applications.

A variety of approaches have been developed for reducing acquisition times by merging the acquisition and estimation steps. These approaches include magnetic resonance fingerprinting [6–8], model-based inverse reconstructions [9–12] or deep-learning methods [13–22] that directly estimate parameter maps from undersampled k-space data. These end-to-end reconstruction techniques can greatly reduce acquisition times, but they require specialized acquisition techniques, and cannot be used to retrospectively process images generated by conventional acquisitions.

In this work we focus on the second part of the conventional processing approach, estimating the transverse relaxation time (*T*_2_) from a series of fully reconstructed magnitude images obtained from a multi-echo acquisition. Estimation is often performed on magnitude images because they are routinely generated by MR scanners and are readily available for both prospective and retrospective studies. The standard technique for this processing, using pixel-wise iterative non-linear least squares (NLLS) fitting, is known to overestimate *T*_2_ when SNR is low [23, 24], because magnitude images have noise with a Rician rather than Gaussian distribution. With Rician noise, the measured signal does not decay to zero at long TEs but rather reaches a plateau that depends on the noise level [23, 25]. Minimizing the square of the difference between the measured and estimated data does not give a maximum likelihood estimation of the parameters, as it would if the noise were Gaussian distributed.

Neural networks (NNs) can be used as an alternative to the NLLS fitting step. Several groups have shown that neural networks (NNs) provide two potential advantages over NLLS fitting: better performance in noisy data, and faster calculation. Prior work using both convolutional neural networks (CNNs) and simpler one-dimensional fully connected neural networks (also called a *multilayer perceptron*) have shown improved noise performance compared to NLLS in diffusion and relaxometry problems [26–32], which both require estimation of exponential decay rates.

In this work, we expand on these prior developments by implementing multiple NN approaches and systematically comparing their quantitative performance for *T*_2_ mapping in the prostate. We propose a novel method for synthesizing large datasets suitable for training and testing networks built from a photographic database. Two synthetic test datasets, one with and one without spatial correlation, are used to isolate the contribution of learned spatial priors to method performance. Networks using 1D and convolutional architectures are implemented, trained, and compared with several conventional fitting methods using synthetic test datasets with known ground truth. Our evaluations focus on performance in low SNR regions, as improved estimation with low SNR data can be used to increase spatial resolution and shorten scan times with higher acceleration factors. Finally, methods are compared on in vivo test data, with and without noise augmentation, to evaluate feasibility and noise robustness.

## Materials and methods

### Signal model

In this manuscript we consider only mono-exponential signal decay using normalized parameters. The signal is described as

1 $$S\left( \eta \right) = S_{0} \exp \left( { - \eta /T} \right),$$

where *η* is the normalized sampling dimension, *T* is the time constant, and *S*_0_ is the signal at *η* = *0*. The sampling dimension is normalized so that the maximum value is 1.0 for a given dataset. This normalization generalizes the problem so that it can describe different sampling times and other mono-exponential processes (e.g., diffusion). For the specific case of *T*_2_ mapping, the echo time TE and the relaxation time constant *T*_2_ are both normalized by the maximum echo time: *η* = TE*/*TE_max_ and *T* = *T*_2_*/*TE_max_.

### Human subjects approval

All human data used in this work were acquired as part of a prospective observational cross-sectional study approved by the University of Minnesota’s Institutional Review Board. The study was originally approved in March 2006 and remains open to accrual as of May 2023. Patients scheduled to receive a clinically indicated prostate MRI exam for the evaluation of suspected prostate cancer were invited to participate in the study, which included research MRI scans in addition to the standard clinical MRI protocol. All patients provided written consent on the day of their exam. In April 2022, a subset of 118 scans with a consistent acquisition protocol and acceptable data quality were selected for this analysis. These 118 datasets were deidentified in compliance with University of Minnesota policy, published on the University’s public data repository, and used for all analyses herein. Note that while the analyses in this manuscript were performed using this deidentified dataset the authors have access to information that could re-identify the participants, as the study is ongoing.

### In vivo MRI acquisition

**Fig. 1:**
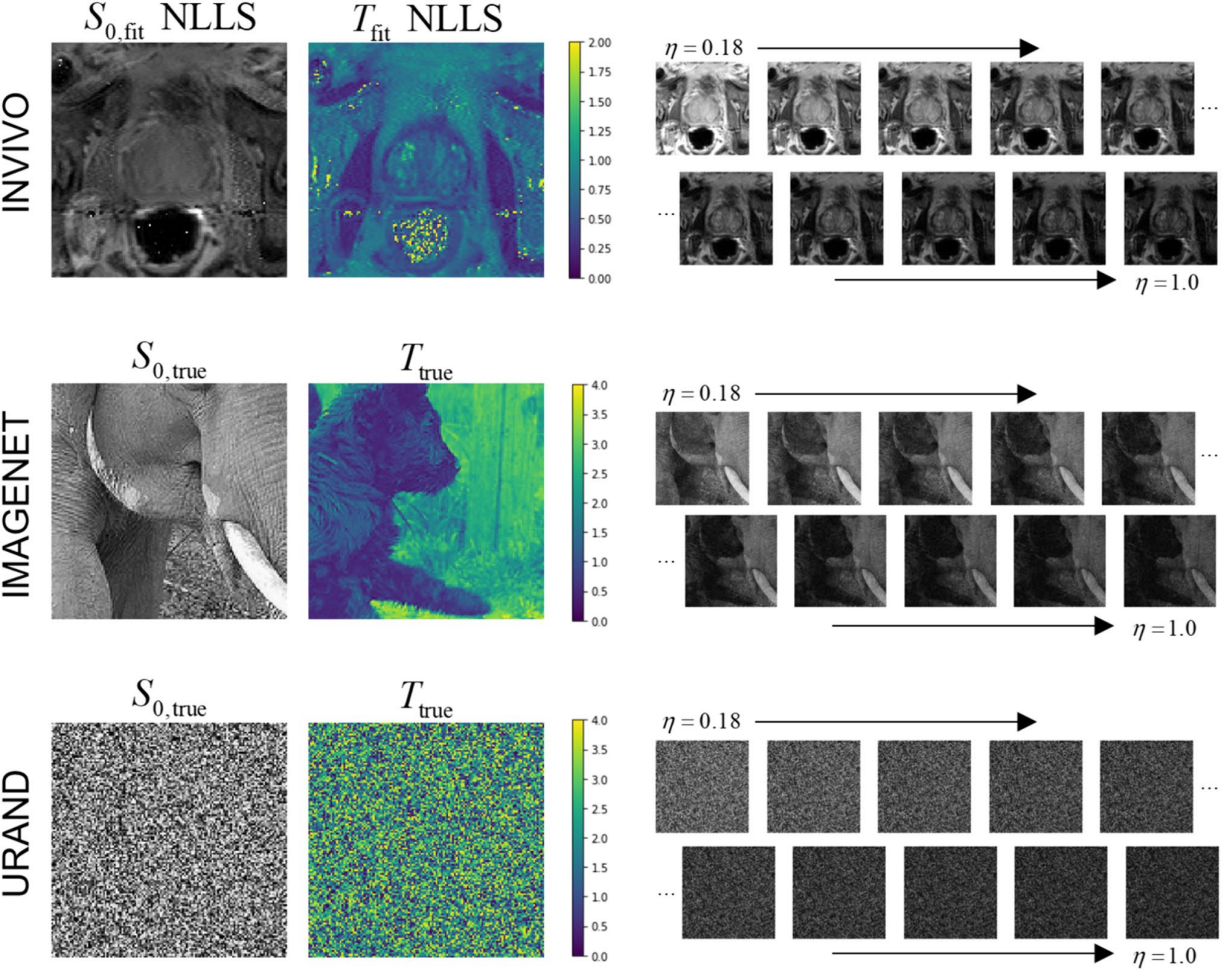
Examples of the three datasets used in the study. The INVIVO data included *T*_2_-weighted images at ten echo points from TE = 26.4—145.2 ms, normalized to *η* = 0.18 to 1.0. The *S*_0_ and *T* maps from conventional NLLS fitting are shown for this example since the true values are unknown. The IMAGENET dataset used photographic images from the ImageNet collection [35, 36] as gold-standard *S*_0_ and *T*, and synthesized exponential image series, with added Rician noise, matching the *η* values from the INVIVO data. The URAND dataset used a uniform random image for both *S*_0_ and *T*, and synthesized an exponential image series in the same manner as for IMAGENET

All prostate MR imaging was acquired on a Siemens 3 T Prisma scanner with surface and endorectal receive coils. Multi-echo multi-slice fast spin-echo MR images were acquired using the vendor’s spin-echo multi-contrast sequence, with parameters TR = 6000 ms, eleven echoes with TE = 13.2–145.2 ms in 13.2 ms increments, 256 × 256 images with resolution 1.1 × 1.1 mm, 19–28 axial slices 3 mm thick, accelerated with GRAPPA *R* = 3, total acquisition time 6.5–8.5 min. Using the normalized signal model of Eq. 1, *η* takes values of 0.091, 0.182, 0.272, …, 1.0. An example image series is shown in Fig. 1.

Each axial slice from these acquisitions was treated as an independent 3D image series, consisting of two spatial dimensions and one *η* dimension. The intensity of each image series was normalized so that the maximum value over all three dimensions was 1.0. Prior to fitting and analyses, each image series was spatially center-cropped to 128 × 128 pixels, and the first echo was discarded to reduce stimulated echo contamination [33, 34]. The full dataset, termed *INVIVO* herein, was randomly split into a test dataset (32 subjects with 695 image series) and a separate training dataset (86 subjects with 1988 image series, not used in this work).

### Synthetic datasets

Two datasets of simulated *T*_2_ relaxometry measurements with known ground truth were generated for training and evaluation. The first dataset, called *IMAGENET* herein, was synthesized from a physics-based signal model using naturalistic images drawn from the publicly available ImageNet dataset [35, 36] as the gold standard values for *S*_0_ and *T*. Using naturalistic images as surrogates for MR images enables the generation of very large datasets, provides a variety of structures and textures, and has been used successfully in training CNNs for MR image reconstruction [37]. For each generated image series, two unique images were selected for *S*_0_ and *T*, converted to floating-point grayscale images, and center-cropped to 128 × 128 pixels. These were scaled so that *S*_0_ ∈ [0,1] and *T* ∈ [0.045, 4], and used to create a series of images *S(η)* from *η* = 0.182, 0.272, …, 1.0 following the signal model of Eq. 1 and matching the in vivo acquisition. Complex Gaussian noise was added to each image, with zero mean and standard deviation *σ* drawn from a uniform random distribution in [0.001, 0.1] for each image series, followed by a magnitude operation. These synthesized images series simulate relaxometry measurements with variable levels of Rician noise, broad parameter ranges, and known ground truth.

The second synthetic dataset, called *URAND*, used random pixel values for the reference images *S*_0_ and *T*. In this dataset, spatially adjacent pixels were randomly drawn, so networks trained on this data could not use neighboring pixels to improve accuracy. This approach was designed as a comparison to the *IMAGENET* approach, with the expectation that networks trained on this data would be less dependent on the statistics of training dataset and less prone to blurring artifacts. This dataset was produced in the same manner as the *IMAGENET* dataset but using gold standard *S*_0_ and *T* images consisting of random pixel values drawn from a uniform distribution over the same ranges.

Examples of image series from all three datasets are given in Fig. 1. For both synthetic datasets, 10,000 image series were generated for *training*, and an additional 1000 image series (with unique *S*_0_ and *T* images and noise) were generated to create independent *test* datasets.

### Curve fitting methods

Four variations of curve fitting were evaluated, which are representative of common practices in quantitative MRI. The FIT_LOGLIN method fit a straight line to the natural logarithm of *S*(*η*) using the *linalg.lstsq()* algorithm in NumPy [38]. This non-iterative linearized method is widely used due to its speed, but the log transformation effectively increases the weighting on the points with higher signal [39]. The results from the FIT_LOGLIN method were used as initial guesses for all other methods.

The FIT_NLLS method used the iterative *optimize.curve_fit()* method of SciPy [40] with the Levenburg-Marquardt algorithm to estimate *S*_0_ and *R* = *1/T* by minimizing the least-squares residuals using Eq. 1. Note *R* was fit rather than *T* to avoid division-by-zero numerical errors. The FIT_NLLS_BOUND method was similar but used the Trust Region Reflective algorithm with bounds *S*_0_ ∈ [0,1000] and *1/T* ∈ [0.25, 22], equivalent to *T* ∈ [0.045, 4], to restrict parameter estimates to physically reasonable values. These two methods, FIT_NLLS and FIT_NLLS_BOUND, assumed a Gaussian noise distribution.

We did not use a true maximum likelihood method with a Rician distribution in this work. This is because our in vivo dataset had spatially varying noise (due to parallel imaging), and in our initial evaluations we found that estimating three parameters (*S*_0_*, R*, *σ*_Rice_) on a per-pixel basis was numerically unstable, likely due to the Bessel functions that describe the Rician distribution. Therefore, we implemented an approximate method that minimized the residual between the measured data and the expectation value of a Rician distribution, as reported by other groups [24, 41]. This method, termed FIT_NLLS_RICE, used *optimize.least_squares()* to fit three parameters (*S*_0_*, R*, *σ*_Rice_*)*, and used the same algorithm and bounds as FIT_NLLS_BOUND. Table 1 summarizes the four fitting methods used in this work.

**Table 1:** Summary of the four curve fitting methods used in this study

Method name	Algorithm	Estimated parameters	Bound parameters
FIT_LOGLIN	Linear regression	*S*_0_, *R*	None
FIT_NLLS	LM, iterative	*S*_0_, *R*	None
FIT_NLLS_BOUND	TRF, iterative	*S*_0_, *R*	*S*_0_, *R*
FIT_NLLS_RICE	TRF, iterative	*S*_0_, *R, σ*_Rice_	*S*_0_, *R*

*TRF *Trust region reflective, *LM *Levenburg-Marquardt

### Neural networks

Two neural network architectures were used in this study. The first was a one-dimensional fully connected neural network used for estimating parameters one pixel at a time. The network had 10 inputs (one for each *η*), 6 hidden layers with 64 weights each, 2 output channels, and used ReLU activations in all layers. The second network was a 2D convolutional neural network based on the enhanced U-Net provided in the MONAI [42] library, which extends the original U-Net [43] with residual units in the first 2 downsampling layers [44]. The network had inputs of 128 × 128 with 10 input channels (one for each *η*), 4 layers of encoding and decoding with increasing widths [128, 128, 256, 512], 3 × 3 convolutions in all layers, outputs of 128 × 128 with two channels (interpreted as *S*_0_, *T*), and used batch normalization and PReLU activations. The NN1D and CNN models had 17,474 and 6.9 M trainable parameters, respectively.

Both networks were trained in a supervised fashion using a mean squared error loss relative to the ground truth *S*_0_ and *T*. Training the 1D networks was performed by loading each image series and iterating over all spatial positions to extract single-pixel decay curves. Training was performed for 5 epochs using a batch size of 10,000 and the AdamW optimizer [45] with learning rate of 0.002. The training sets used 800 image series (thus 128*128*800 = 13.1E6 1D series) for training and 200 image series (3.3E6 1D series) for validation. Convolutional networks were trained for 1000 epochs with a batch size of 100 image series and an AdamW optimizer with learning rate =0.002. Each training set was further divided into train (80%) and validation (20%) subsets for monitoring training progress. Table 2 summarizes the four NN models trained in this work.

**Table 2:** Summary of the four NN models trained and used in this study

Model name	Architecture	Training data
NN1D_IMAGENET	NN1D	IMAGENET
NN1D_URAND	NN1D	URAND
CNN_IMAGENET	CNN	IMAGENET
CNN_URAND	CNN	URAND

### Evaluation on synthetic datasets

Comparisons between all eight parameter estimation methods (four fitting, four NNs) were performed by evaluating all methods on the two synthetic test datasets (IMAGENET and URAND), which allowed for analysis of error because the ground truth *T* maps are available. Each method was used to estimate a *T* map for each image series in the test datasets. The signed error between true and estimated maps *(T*_pred_ − *T*_true_), and the absolute error (|*T*_pred_ − *T*_true_|), were calculated for each pixel. Errors were summarized on a per-slice basis by taking the median error value over each map. The median value of the signed error was interpreted as the **bias** of a method. The interquartile range (IQR, 75th–25th percentile) of the signed error was interpreted as a measure of **precision**. The median value of the absolute error was interpreted as a measure of overall **accuracy**, which is dependent on both bias and precision, with smaller errors indicating higher accuracy. Decomposing accuracy into separate contributions of bias and precision is important for understand potential sources of bias in quantitative analyses [46, 47].

Errors were also evaluated on a per-pixel basis to assess their dependence on SNR and *T*. SNR was calculated on a per-pixel basis using

2 $${\text{SNR}}_{{{\text{dB}}}} = 20\log_{10} \left( {\frac{{\left\| {S\left( \eta \right)} \right\|_{2} }}{\sigma \sqrt N }} \right),$$

where *σ* is the standard deviation of noise, *N* is the number of *η* values, and ||…||_2_ is the L2-norm. With this definition the SNR of the synthetic data varied spatial and ranged from negative infinity (where *S*_0_ = 0) to 59 dB (for *S*_0_ = 1, *T* = 4, *σ* = 0.1), covering a realistic range of in vivo values. Finally, each predicted *T* map was compared to the true *T* map using the structural similarity index measure (SSIM) [48], a quantitative measure of perceptual similarity between two images, to evaluate the suitability of the method for producing subjectively interpretable *T* maps.

### Evaluation on in vivo dataset

The best performing methods from the synthetic evaluation were subsequently evaluated on the INVIVO test dataset. We compared the results of each method to the results of the FIT_NLLS method because there is no true *T* map available, and FIT_NLLS represents the most conventional approach. Using the SNR definition of Eq [2] and estimating *σ* with the per-pixel residual from the FIT_NLLS fitting, the SNR of the INVIVO test dataset averaged 22.2 dB, with range from 0 to 42.7 dB (99.99% percentile). The *T* maps were compared qualitatively to determine if their estimation performance was consistent with the findings in the synthetic experiment.

Finally, a noise-addition experiment was performed to evaluate the sensitivity of each method to progressively increasing noise. Complex Gaussian noise with standard deviation ranging from *σ* = 0.02–0.08 units was added to each normalized image series in the INVIVO test dataset, followed by a magnitude operation to generate an image series with increased Rician noise. Original and noise-augmented datasets were used as input to estimate *T* maps using all the methods, without retraining any NNs. Each map was compared to the *T* map generated from the original (no added noise) image series using the same method, so that the per-slice error was the median over *T*_added_noise_ − *T*_original_. Bias, precision, and accuracy with increasing noise levels were interpreted in the same manner as in the synthetic evaluation.

All computation for this work was performed in Python using the PyTorch [49] and MONAI [42] libraries on a Linux workstation with an AMD 7950X CPU, 128 GB RAM, and a single Nvidia RTX 4090 GPU. All data, code, and trained models used in this work have been made publicly available (see *Data Availability*, below).

## Results

### Computing performance

Training the NN1D methods required 220–240 s per epoch, requiring ~20 min of training. The CNNs used ~40 s per epoch, for a total training time of ~11.1 h for each dataset. Inference was fastest with the small NN1D networks, requiring an average of ~25 ms per case on the INVIVO test data, while the CNNs inference time was ~138 ms per case. The fitting methods were slower, as expected requiring 233 ms per case for FIT_LOGLIN, 551 ms for FIT_NLLS, 1.6 s for FIT_NLLS_BOUND, and 3.5 s for FIT_NLLS_RICE.

### Performance on synthetic datasets

**Fig. 2:**
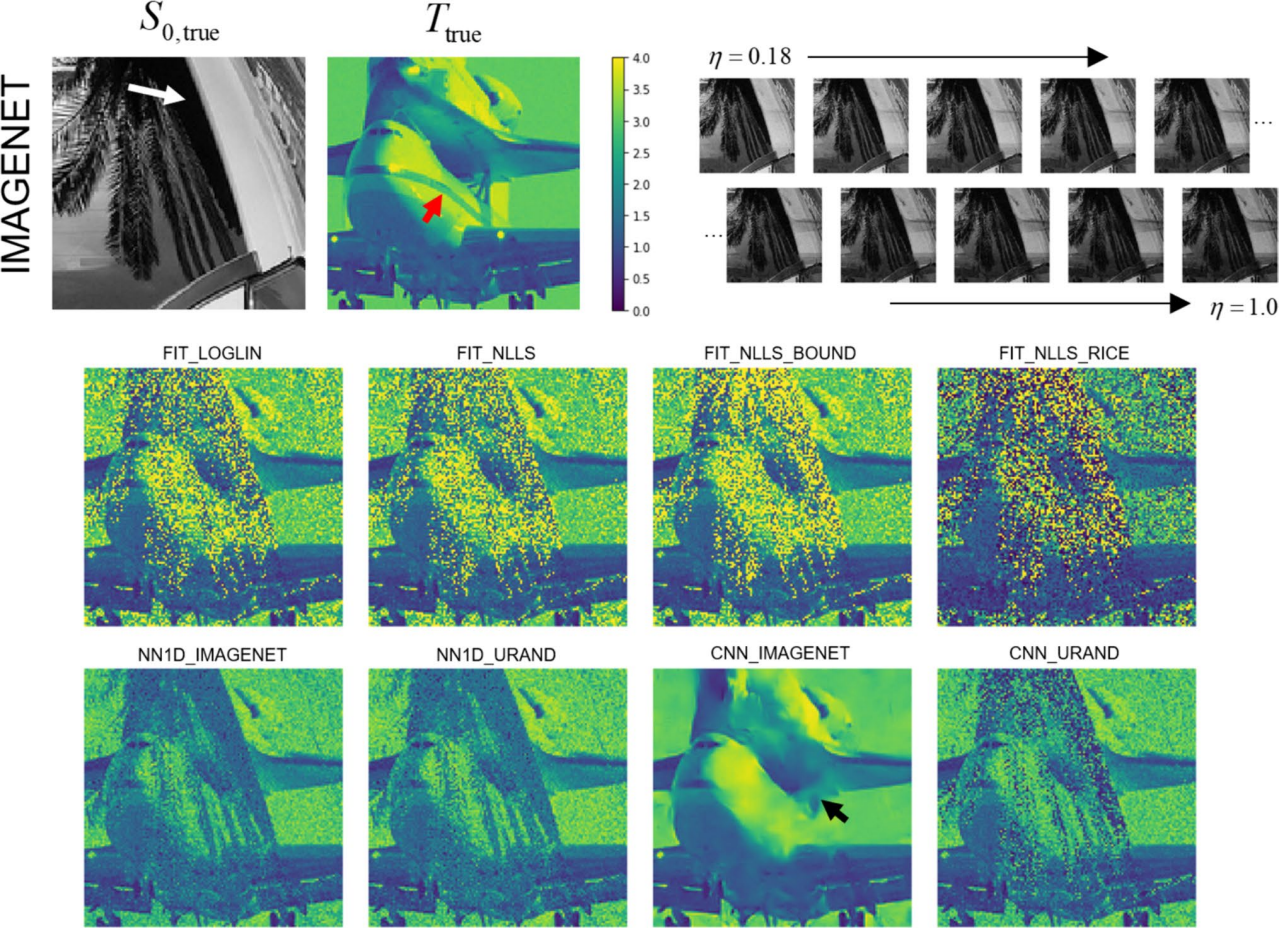
Example showing estimated *T* maps from all 8 methods for a case from the synthetic IMAGENET test dataset. The true *S*_0_ and *T* maps are shown above. The regions of low *S*_0_ values (e.g., white arrow) have low SNR, which can be seen as regions of incorrect values in each of the estimated *T* maps. This example shows how the methods vary in performance at various noise levels and how they fail in regions of very low SNR. In this example CNN_IMAGENET had the smallest MAE (0.09) compared to the conventional FIT_NLLS (MAE = 0.31) but shows evidence of blurring (black arrow) in the low SNR regions. Note also that none of the methods recovered the stripe visible in the *T*_true_ map (red arrow), underscoring the difficulty of this inverse problem

All eight parameter estimation methods were evaluated on all image series in the IMAGENET and URAND test datasets. An example image series from the IMAGENET test dataset is shown in Fig. 2, with *T* maps estimated by all methods. In the high-SNR regions, most methods produced similar-appearing results, although CNN_IMAGENET had distinctly lower noise. The estimated *T* maps were most different in the low-SNR regions, where the fitting methods showed high noise levels, whereas CNN_IMAGENET better recovered a noise-free *T* map, at the cost of modest blurring and distortion.

**Fig. 3:**
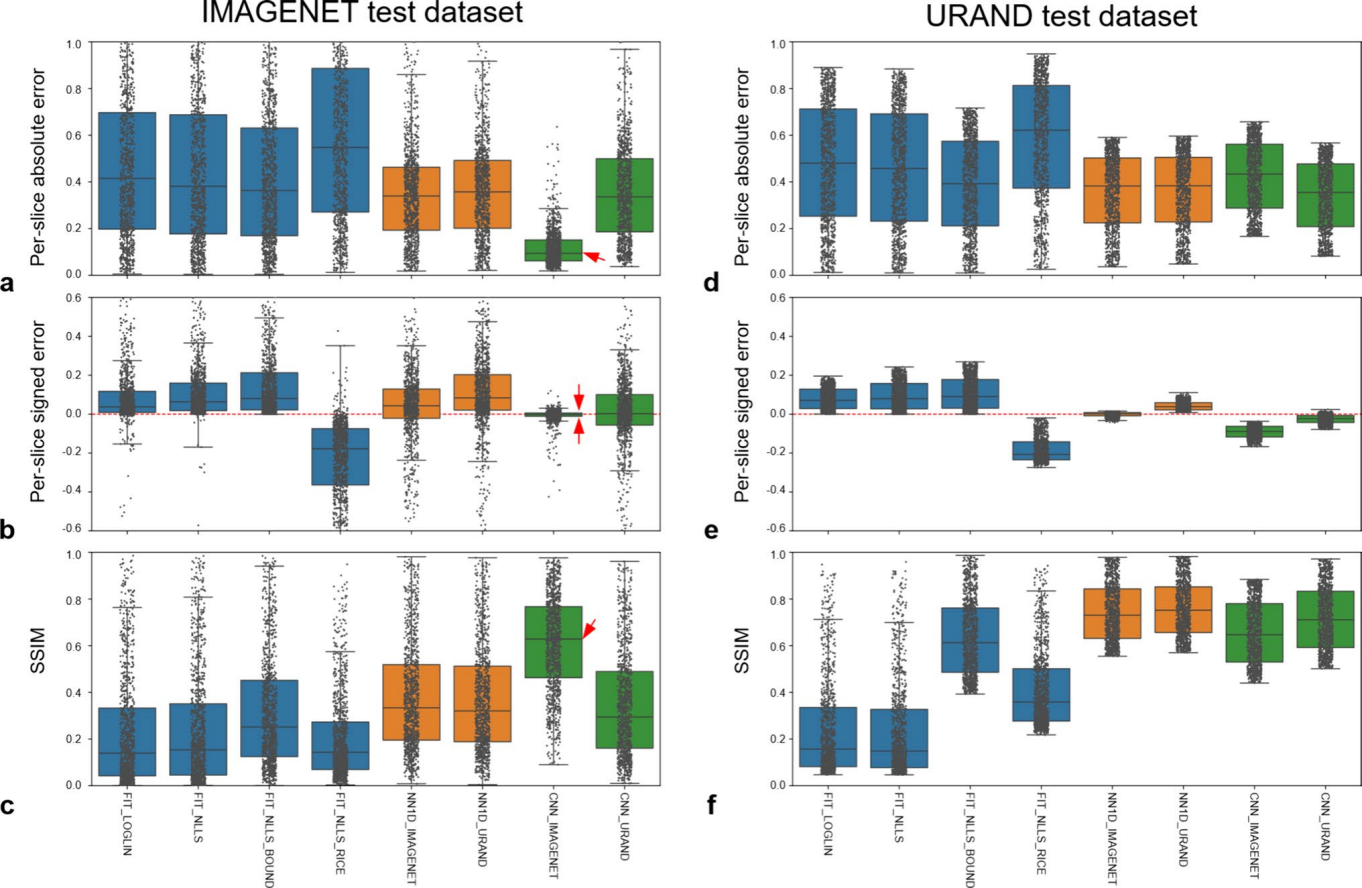
Comparative performance of the 8 methods. Quantitative evaluations of the estimated *T* map are shown for the synthetic IMAGENET (left column) and URAND (right column) test datasets, each consisting of 1000 image sets. The top row (**a**, **d**) plots the per-slice the absolute error (|*T*_pred_ − *T*_true_|), the middle row (**b**, **e**) plots the signed error (*T*_pred_ −* T*_true_), and the bottom row (**c**, **f**) plots the structural similarity between *T*_pred_ and *T*_true_. All box-whisker plots show median, interquartile ranges (IQR), and extrema (>1.5*IQR from quartiles), overlaid with the values from each of the 1000 cases. On the IMAGENET dataset, CNN_IMAGENET gave the lowest overall error (panel **a**), very low bias and highest precision (panel **b**), and the highest SSIM (panel **c**), as indicated by red arrows

Figure 3 provides a quantitative comparison of the methods’ performance in estimating *T* maps on both synthetic datasets. Focusing first on the four fitting methods, the two most common approaches (FIT_LOGLIN, FIT_NLLS) performed similarly, and exhibited a positive bias as expected. Incorporating bounds to the fitting (FIT_NLLS_BOUND) provided a small improvement of overall accuracy (i.e., reduced absolute error). The FIT_NLLS_RICE method had poorer accuracy and precision, likely due to the need to estimate three parameters instead of two.

Looking at the NN methods, the performance of CNN_IMAGENET on the IMAGENET test dataset stands out, showing low bias and the highest overall accuracy, precision, and structural similarity with the reference *T* maps. Notably, this method did not perform as well on the URAND test dataset. More generally, the fitting and NN1D methods performed similarly on the IMAGENET and URAND datasets, whereas CNNs gave higher accuracy on the IMAGENET dataset. This difference in performance suggests that, as expected, the CNNs use the information from spatially adjacent pixels to improve performance; when applied on data without spatial correlation the performance decreases.

**Fig. 4:**
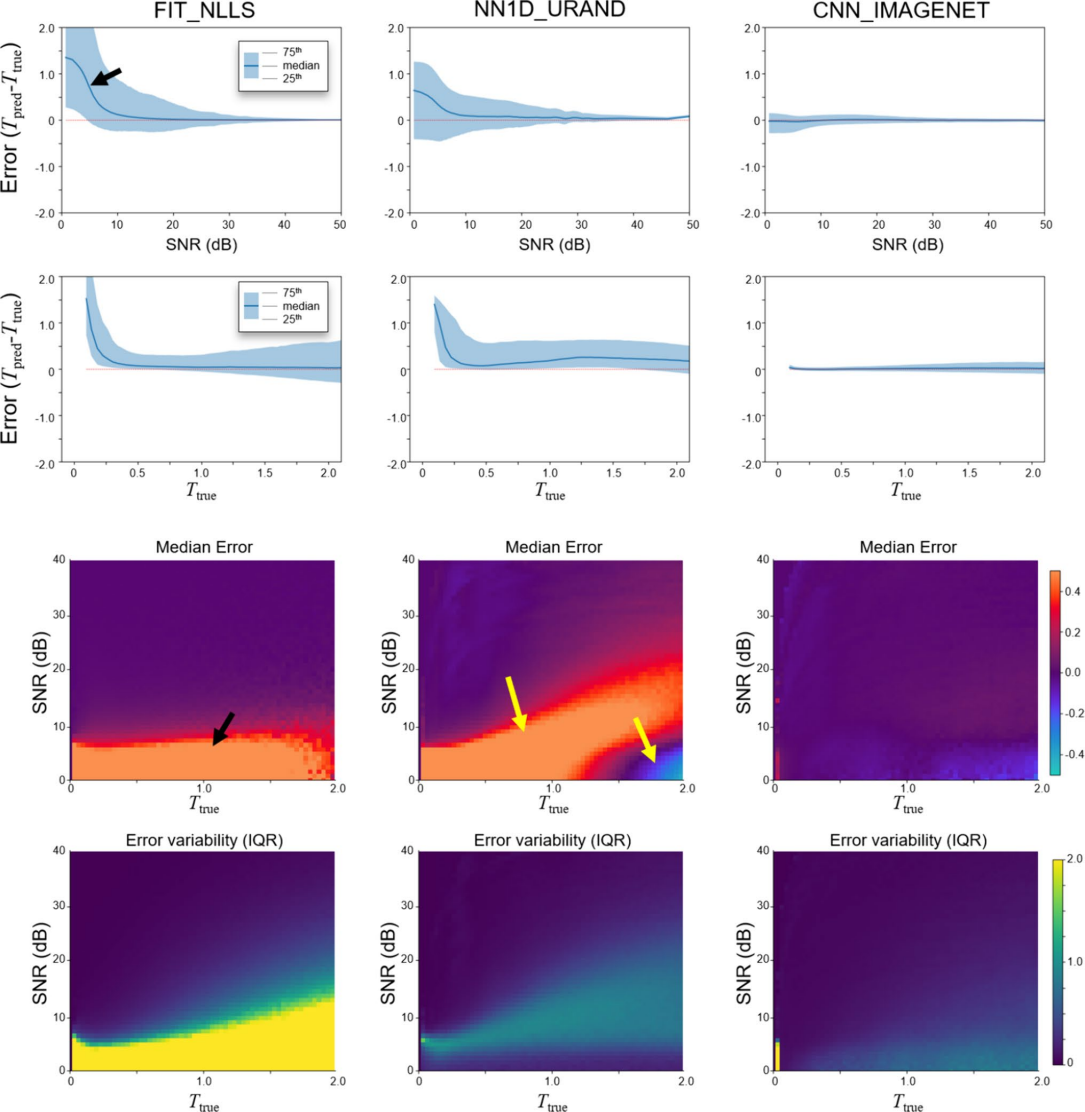
Estimation error (*T*_err_ = *T*_pred_ −* T*_true_) as a function of *T*_true_ and SNR. Plots are shown for the three selected methods, on the IMAGENET test dataset, evaluated on a per-pixel basis. Both *T*_true_ and SNR dimensions were divided into 100 bins, and the median value and IQR (25th and 75th percentile) were calculated for each bin. Top row shows *T*_err_ as a function of SNR, while the second row shows the dependence on *T*_true_. The bottom two rows plot the median and IQR of *T*_err_ as a function of both *T*_true_ and SNR to show the interplay of these two effects. The FIT_NLLS method shows positive bias at low SNR (black arrows), while CNN_IMAGENET has low bias and variability throughout. NN1D_URAND shows both over- and underestimation of *T* at low SNR (yellow arrow)

For brevity, a subset of methods that performed well on the IMAGENET dataset of Fig. 4 were selected for subsequent analyses: the conventional FIT_NLLS, the best performing CNN_IMAGENET, and the NN1D_URAND method, as these both offered good performance and represent distinctly different methodologies.

Figure 4 presents the same experiment as Fig. 3 but analyzed on a per-pixel basis rather than per-slice, in order to evaluate errors as a function of SNR and *T*_true_ values. The FIT_NLLS results (first column) demonstrate the tendency of this method to overestimate *T* when SNR is at low levels. CNN_IMAGENET showed low variability and bias with relative consistency across values of SNR and *T*_true_, which are highly favorable characteristics for a *T* estimating method. NN1D_URAND had inconsistent behavior, both over- and underestimating *T* in different regimes of SNR and *T*_true_. Note that at high SNR, FIT_NLLS and CNN_IMAGENET gave similar performance, with low error and variability.

### Performance on in vivo dataset

**Fig. 5:**
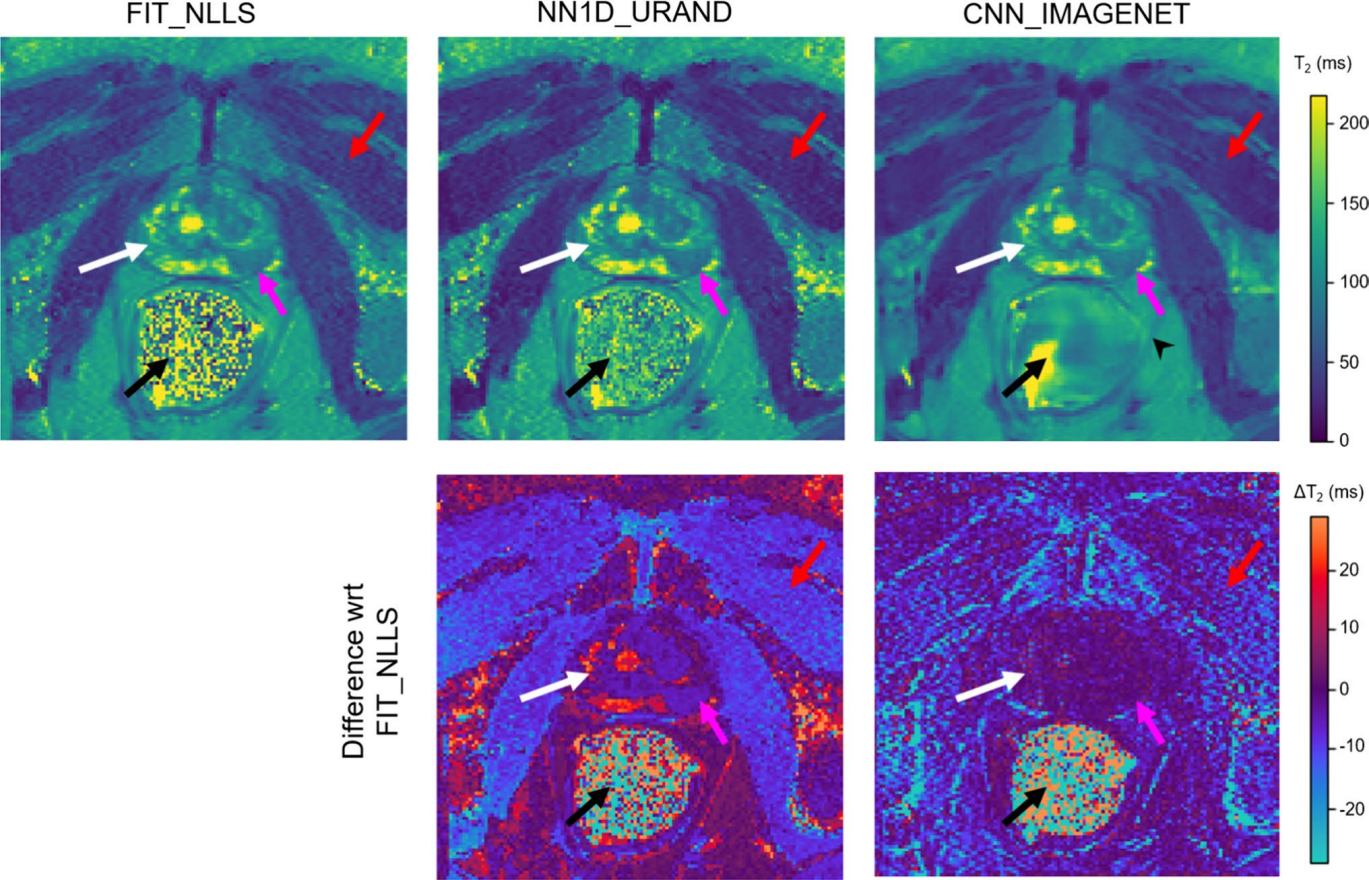
Comparison of selected methods on a slice containing a histologically-confirmed cancer (left-side peripheral zone, magenta arrow) from the INVIVO test set. Since no true value of *T*_2_ is available, the methods were compared to the standard FIT_NLLS. The top row shows *T*_2_ maps from each method; the second row shows the signed difference relative to FIT_NLLS. In the prostate (white arrow), where the SNR is high, CNN_IMAGENET values were similar to FIT_NLLS, whereas NN1D_URAND gave *T*_2_ estimates that were more variable. In the low SNR muscle region (red arrow) results were less consistent, and in the rectum region (black arrow, where SNR is 0 due to a perfluorocarbon-filled balloon, CNN_IMAGENET shows reconstruction artifacts. The CNN_IMAGENET result appears least noisy, but shows blurring in some regions (e.g., black arrowhead)

An example comparing the four selected methods on an INVIVO test case is given in Fig. 5. This case is a mid-prostate slice from a 65-year-old male with a peripheral zone lesion (magenta arrow) determined to be a Grade Group 3 (Gleason 4 + 3) cancer on subsequent targeted biopsy and prostatectomy. The *T*_2_ maps from the three methods generally look similar, but CNN_IMAGENET shows less noise overall and some blurring in the lowest SNR regions. All methods give similar *T*_2_ values in the lesion (88.1 ms for FIT_NLLS, 82.7 ms for NN1D_URAND, 87.7 ms for CNN_IMAGENET), which has high SNR. Differences between the methods can be more clearly seen by separately focusing on regions with high (prostate, white arrow) and low (muscle, red arrow) SNR. CNN_IMAGENET gave similar values to FIT_NLLS in high-SNR regions, but lower values in low SNR regions, and shows some blurring in the lowest SNR regions. Additional plots comparing the *T*_2_ values for this case by region are provided in Supplemental Fig S1. All of these relationships seen in this case are consistent with the results shown in the synthetic data. This suggests that the observations from the synthetic data apply to the in vivo case: CNN_IMAGENET gives accurate *T* estimates independently of *T* and SNR, whereas FIT_NLLS overestimates *T* in low SNR regions but gives accurate results where the SNR is high.

**Fig. 6:**
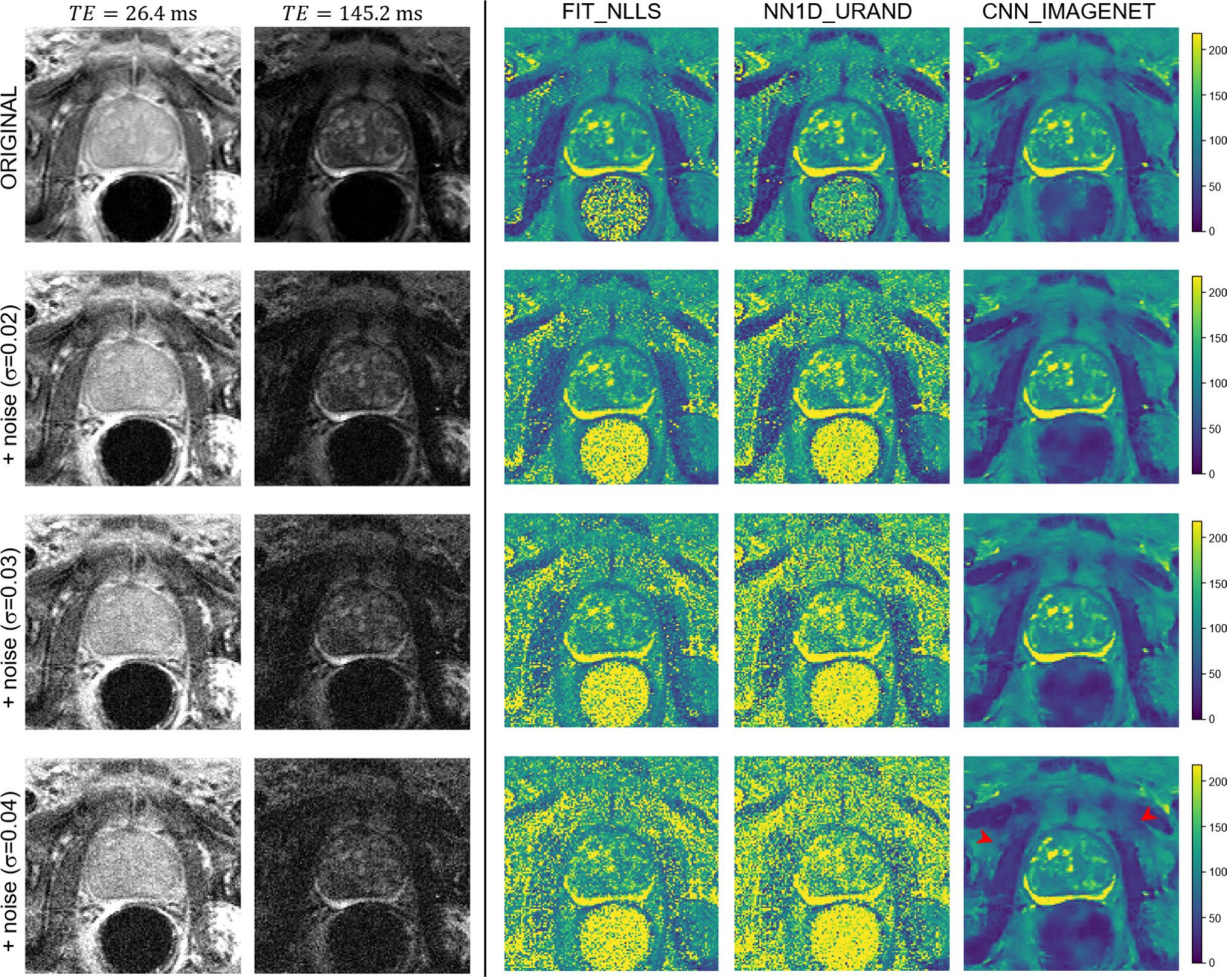
An example in vivo case from the noise-addition experiment. No cancer is present in this slice based on subsequent biopsy. The top row shows the unmodified data: images of the shortest and longest echo time, and *T*_2_ maps calculated with the four selected estimation methods. The next three rows show the same data with increasing noise added (with Gaussian standard deviation 0.02, 0.03, and 0.04), and the corresponding *T*_2_ maps. At higher levels of added noise, the predicted *T*_2_ maps from FIT_NLLS and NN1D_URAND show increasing noise and increased higher values of *T*_2_ throughout the image. In contrast, CNN_IMAGENET shows modest blurring (red arrowheads) and no noise amplification with increasing added noise

**Fig. 7:**
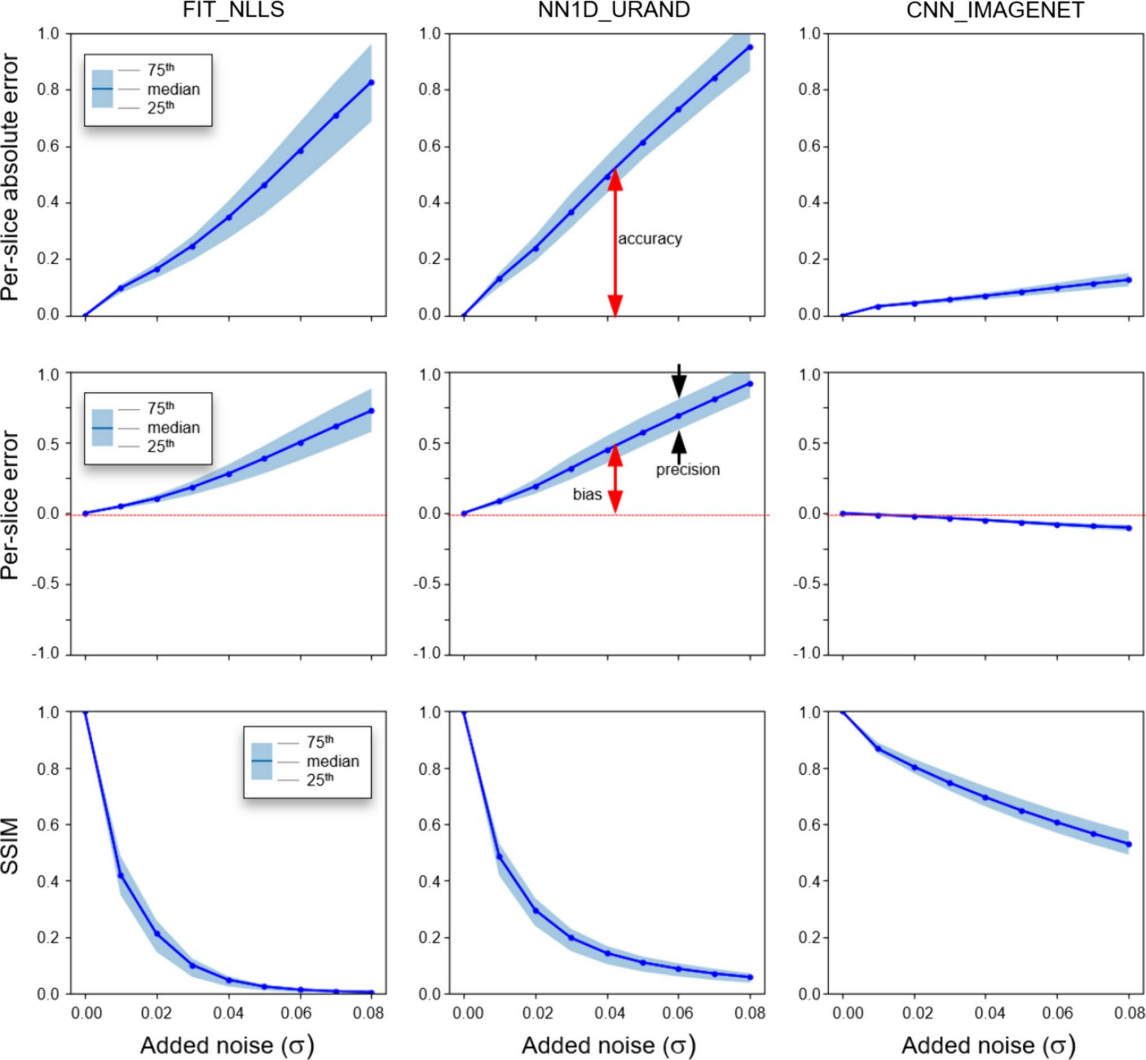
Results from the noise-addition experiment. Plots show the per-slice median absolute error (top row), per-slice signed error (middle row), and SSIM relative to the reference *T*_2_ map, which is the map calculated using the same method on the original (no noise added) dataset. Plots show the median and IQR of all 694 slices in the INVIVO test set as the level of added noise increases from left to right. Annotations indicating definitions of bias, precision, and accuracy are included for clarity. CNN_IMAGENET shows the least change in these three metrics with increasing added noise

The results of the noise addition experiment, shown in Figs. 6 and 7, also support this interpretation. Figure 6 shows an example case from the INVIVO test dataset and the estimated *T* map from the three methods, with increasing amounts of retrospectively added Rician noise. The FIT_NLLS method showed increasing variation and increasing values for *T*_2_ at higher noise levels. In contrast, the CNN_IMAGENET showed consistent values of *T*_2_, but moderately increased blurring in the *T*_2_ maps. These trends can be seen across all cases in the INVIVO test dataset, as shown in Fig. 7. The NN1D_URAND model performed similarly to FIT_NLLS, but the CNN_IMAGENET model showed greater robustness to noise, with smaller changes in accuracy, bias, precision, and SSIM relative to the *T*_2_ maps calculated from the original (no noise added) data. A prospectively acquired dataset shown in the supplemental Fig S2 and S3 shows similar results as this retrospective case, with CNN_IMAGENET giving better noise robustness than the other methods.

## Discussion

In this work, we compared several methods for estimating *T*_2_ from prostate *T*_2_ relaxometry acquisitions. Our main finding is that a CNN, trained in a supervised fashion with a physics-based synthetic dataset (CNN_IMAGENET), gave the best overall accuracy when tested on simulated data. Additionally, when evaluated on in vivo data the performance was similar to that of the simulated evaluation, giving values in agreement with conventional NLLS fitting in the high SNR regime, and providing better precision and a likely reduction in bias in low SNR regions.

Importantly, the comparison amongst the NN variations and four fitting techniques provides insight into three critical factors that enable CNN_IMAGENET to outperform the conventional NLLS approach. The first factor is the ability of the synthetic training strategy to correctly incorporate the Rician noise distribution. While the problem of least-squares fitting in Rician noise is widely recognized [24, 41], the solutions proposed require knowledge of the noise distribution on a per-pixel basis, which is not easily determined retrospectively from images reconstructed with parallel imaging. Parallel imaging is routinely used to reduce acquisition times but produces images with a spatially varying noise distribution. Trying to estimate the pixel-wise noise simultaneously with the relaxation rate increases the degrees of fitting of the model and leads to additional error and bias in the other parameter estimates. The Rician noise issue is a consequence of the specific problem domain we chose to focus on: fitting previously reconstructed magnitude images with spatially varying noise. This issue can be avoided by fitting real- or complex-valued data [50, 51], or by generating accurate noise maps from calibration acquisitions, but these are not generally output from MR scanners and can be sensitive to phase errors. Our focus on magnitude images has broad practical value: it allows these methods to be used for retrospective analyses of conventionally acquired relaxometry datasets available in DICOM format, and it can be more readily applied to clinical studies without needing custom pulse sequences and reconstructions.

The second factor contributing to improved quantitative performance is that NNs trained in a supervised fashion produce outputs that are limited by the range of values present in the training data. An unconstrained NLLS fit can lead to a very large range of parameter estimates, particularly in noisy data. Using a constrained fit (FIT_NLLS_BOUND) with relatively wide limits substantially reduces the error compared to unbound fitting. The synthetic datasets used for training had values of T drawn from a uniform random distribution over the same range as the bounds in FIT_NLLS_BOUND (*T* ∈ [0.045, 4]), so these methods had similar range constraints.

The distribution of parameters in the synthetic training data limits the range of values that are produced by NN inference, but they can also bias the output values. This bias, called a domain or distribution shift [52], can be avoided by careful selection of the parameter distributions in the training set. In *T*_2_ mapping, the TE array is generally selected to cover the range of *T*_2_s the investigators anticipate. The bias of expected *T*_2_ values is essentially built directly into the acquisition parameters. By synthesizing training data with a uniform distribution of *T*_2_ values, over a wide range (from 0.25 times the shortest TE value to 4 times the largest), the negative impact of bias is minimized. For the parameters used in this study, the *T*_2_ values used in the training set were uniformly distributed from 6.6 to 580.8 ms, which includes the full range of *T*_2_s reported in healthy prostatic tissue and cancers (roughly 40–400 ms [1–3, 5]) with a wide margin. Similarly, the SNR of the synthetic training data (0–59 dB) was designed to be substantially wider than that of the targeted INVIVO test set (0–43 dB) to avoid a bias from distribution shift.

The third factor is the convolution operator, which takes advantage of the spatial correlation between pixels and improves performance in low SNR regions. The training strategy used for CNN_IMAGENET, in which the network attempts to predict noise-free *T* maps from noisy image series, provides inherent denoising, as the model implicitly “learns” the Rician distribution and seeks to remove it. The spatial convolution operator is a key component of this denoising process—we showed that a 1D network trained on the same data (NN1D_IMAGENET) exhibited lower overall precision (Fig. 4) and greater estimation variance at low SNR levels.

Improvement in noise robustness comes at the cost of some blurring, which can be seen in our data as well as in prior studies [32, 53]. The amount of blurring depends on the architecture, training strategy, and loss functions. Zhao et al. [54] attribute this phenomenon to the use of an L2 loss function, and have proposed alternate loss functions that could improve the perceived image quality. Improving these artifacts, while maintaining quantitative performance, is a topic for future work.

The strategy of synthetic supervised training, using a large synthetic dataset derived from a physics-based signal model, has broad applications in the MR field. As first demonstrated with the AUTOMAP image reconstruction method [37], this approach can be used to build large datasets for training, and can encode signal models with much greater complexity than the simple monoexponential decay shown here. The dataset synthesis encodes the forward signal model, and through the training process the network learns a mapping that encodes the inverse signal model. In this work we demonstrated its application in prostate *T*_2_ relaxometry, but this same strategy can be used for other relaxometry applications, diffusion modeling, and potentially problems with higher-dimensional signal models.

In addition to improving retrospective analyses, the methods demonstrated herein could be used to optimize acquisitions for prospective studies. With increased robustness to low image SNR, it may be possible to acquire data with higher accelerations or spatial resolution without losing quantitative performance. Furthermore, since the CNN_IMAGENET method has lower variability than FIT_NLLS in estimating *T*_2_ at long values (e.g., *T*_2_ > TE_max_, or *T* > 1, as shown in Fig. 5), it may be possible to acquire shorter echo trains to reduce heating (specific absorption ratio) and/or increase spatial resolution within the same scan time. An example demonstrating this approach for increasing spatial resolution is provided in supplemental Figs. S2 and S3; future work will explore the potential for acceleration and improving spatial resolution in prospective acquisitions.

Similarly, the diagnostic performance of *T*_2_ mapping for distinguishing malignant lesions (low *T*_2_) from benign and normal tissues (longer *T*_2_) would also depend on image SNR. With sufficiently high SNR, conventional methods (FIT_LOGLIN and FIT_NLLS) and CNN_IMAGENET give the same *T*_2_ values, and thus would give the same diagnostic performance. At low SNR, the conventional methods will overestimate *T*_2_, and the effect would be greater for shorter *T*_2_ values. This would effectively decrease the measured dynamic range of *T*_2_ in the prostate by overestimating the values in low-*T*_2_ regions like malignant lesions and having less effect on the longer *T*_2_s found in benign and healthy tissues. The use of noise-robust parameter estimation like that provided by CNN_IMAGNET may enable prospective acquisitions with lower SNR with less impact on the diagnostic specificity of *T*_2_.

This study has several limitations. First, the assumption of a monoexponential decay curve for *T*_2_ relaxometry is an approximation. While discarding the first point of a multi-echo dataset reduces much of the error associated with stimulated echoes [33, 34], fitting the data to echo modulation curves from Bloch simulations could give better accuracy and correct B_1_ and slice profile effects [55]. Future work will extend our proposed approach by using Bloch simulations to generate the synthetic data used to train the NNs. Second, this work used relatively simple NN architectures and training strategies, which could be improved upon using architectural variations, different loss functions, and expanded or augmented training datasets. A disadvantage of the model architectures used is that they are sized and trained to work only for a specific set of *η* values; to apply this approach to data with different TE values, one would need to generate a new synthetic dataset and train a new model for that specific set of parameters. Third, we did not compare our methods with Bayesian fitting [56, 57] or dictionary-based parameter estimation, two additional strategies that merit further exploration. These methods could incorporate Rician noise and restricted parameter ranges but would not easily incorporate the learning of spatial priors provided by CNNs. Finally, we did not evaluate the performance on in vivo data from multiple sites, vendors, or field strengths. Since no information about these factors was used in generating the synthetic training data, the performance may extend to heterogeneous datasets, but this assessment was not performed.

## Conclusion

We compared conventional NLLS fitting with several neural network architectures and training strategies for estimating *T*_2_ maps from multi-echo magnitude images acquired for prostate relaxometry. We found that a CNN, trained with synthetic data in a supervised manner, gave better accuracy and noise robustness than NLLS fitting and other NN methods. By comparing the performance of different estimation methods on multiple synthetic datasets we were able to identify three specific factors that led to the performance gains: improved Rician noise modeling, restriction of the parameter estimation domain, and learning of spatial priors with convolutional layers. Furthermore, we showed the feasibility of using these CNNs for analyzing in vivo prostate *T*_2_ relaxometry data and demonstrated its excellent performance in the low SNR regime.

## Supplementary Information

Below is the link to the electronic supplementary material.

Supplementary file1 (DOCX 4045 KB)

## Data Availability

All of the de-identified in vivo data used in this study are available in the Data Repository for the University of Minnesota, at https://conservancy.umn.edu/handle/11299/243192 [58]. The source code for training neural networks, evaluating results, and generating the figures in this manuscript, along with the trained models, are available on Github at https://github.com/patbolan/prostate_t2map.

## Abbreviations

CNN: Convolutional neural network
IQR: Interquartile range
NLLS: Non-linear least squares
NN: Neural network
SNR: Signal-to-noise ratio
SSIM: Structural similarity index measure
