# Supplementary material for: Improved quantitative parameter estimation for prostate T2 relaxometry using convolutional neural networks
Source: MAGMA. 2024 Jul 23;37(4):721–35. doi: 10.1007/s10334-024-01186-3 (PMC11417079; doi:10.1007/s10334-024-01186-3)
Supplement: Supplementary file 1 — Supplementary file1 (DOCX 4045 KB) [file 10334_2024_1186_MOESM1_ESM.docx]

## Supplemental Data


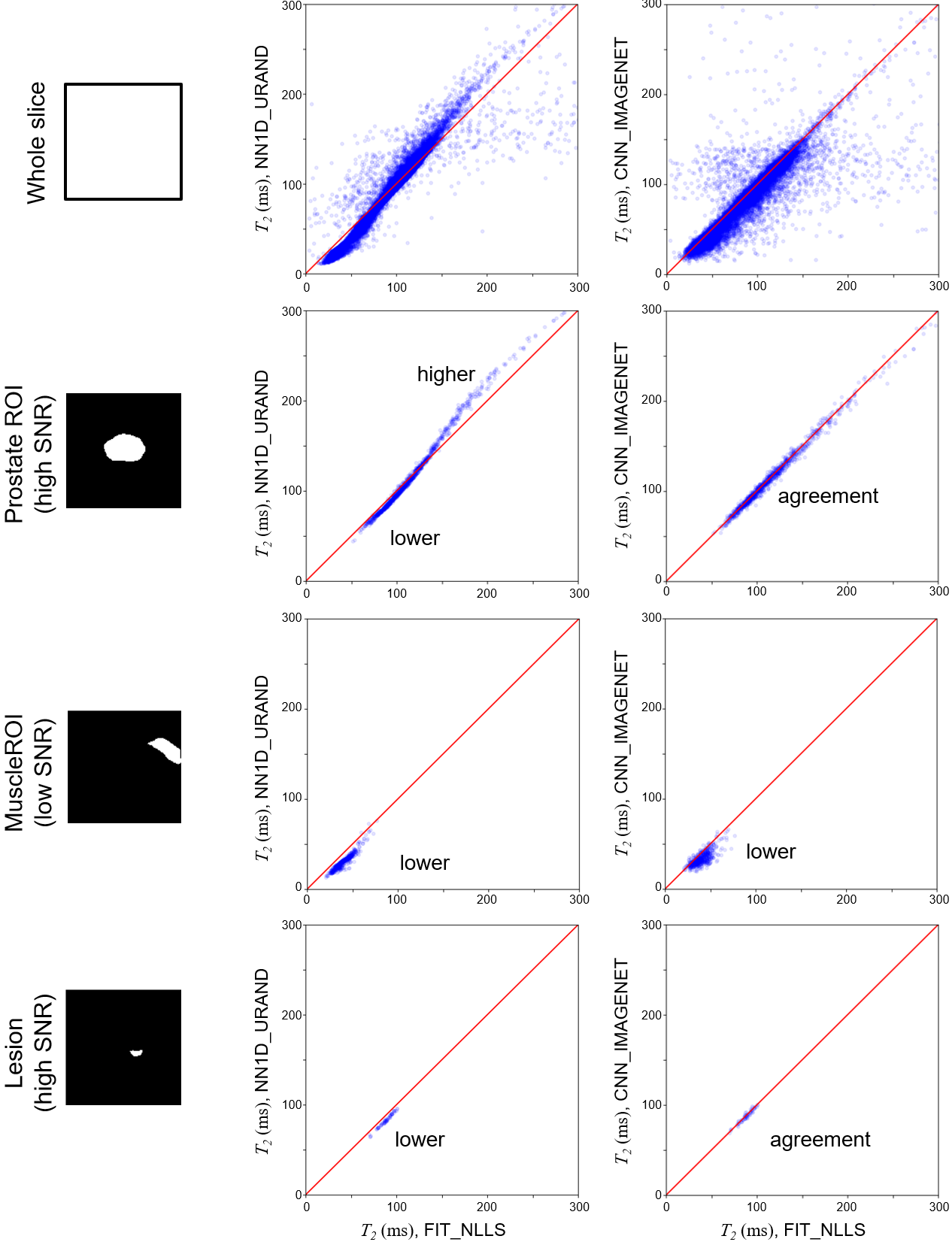


Fig S1 – A pixelwise comparison of T_2_ values from the example of Fig 5, separated by different regions. In the high SNR ROIs (whole prostate, lesion), CNN_IMAGENET gives values similar to FIT_NLLS, but in the low SNR ROI (muscle) CNN_IMAGENET gives lower values. Comparing this trend with the synthetic data of Fig 5 suggests that the CNN_IMAGENET values are correct, and FIT_NLLS overestimates T2 in low SNR regions. NN1D_URAND gives both lower and higher values than FIT_NLLS, depending on the range of T_2_.


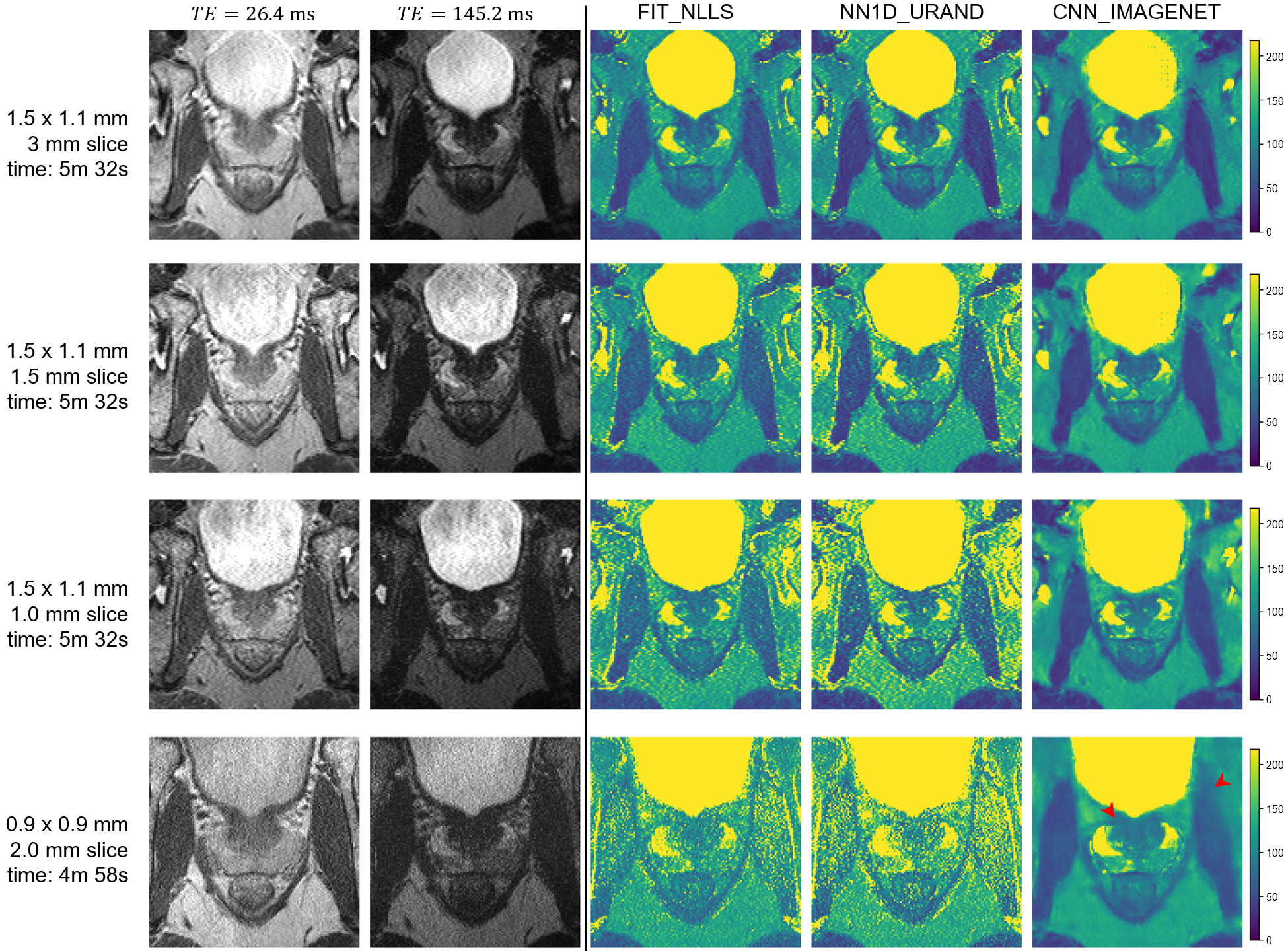


Fig S2 – A prospectively acquired dataset from a healthy volunteer acquired with progressively decreasing SNR. Similar to the retrospective noise addition study of Figs 6 and 7, the top row shows the standard acquisition and associated T_2_ maps acquired with 3 mm slice thickness. The middle two rows show the same scan with the slice thickness decreased to 1.5 mm and 1.0 mm; the final row shows higher resolution measurement with a 2 mm slice but higher in-plane resolution. As in the noise addition experiment, the T_2_ maps from CNN_IMAGENET show lower noise levels, but some blurring in regions where the SNR is very low (red arrowheads). This example demonstrates how a noise-robust quantitative parameter estimation method can be used prospectively to increase spatial resolution without increasing acquisition time.


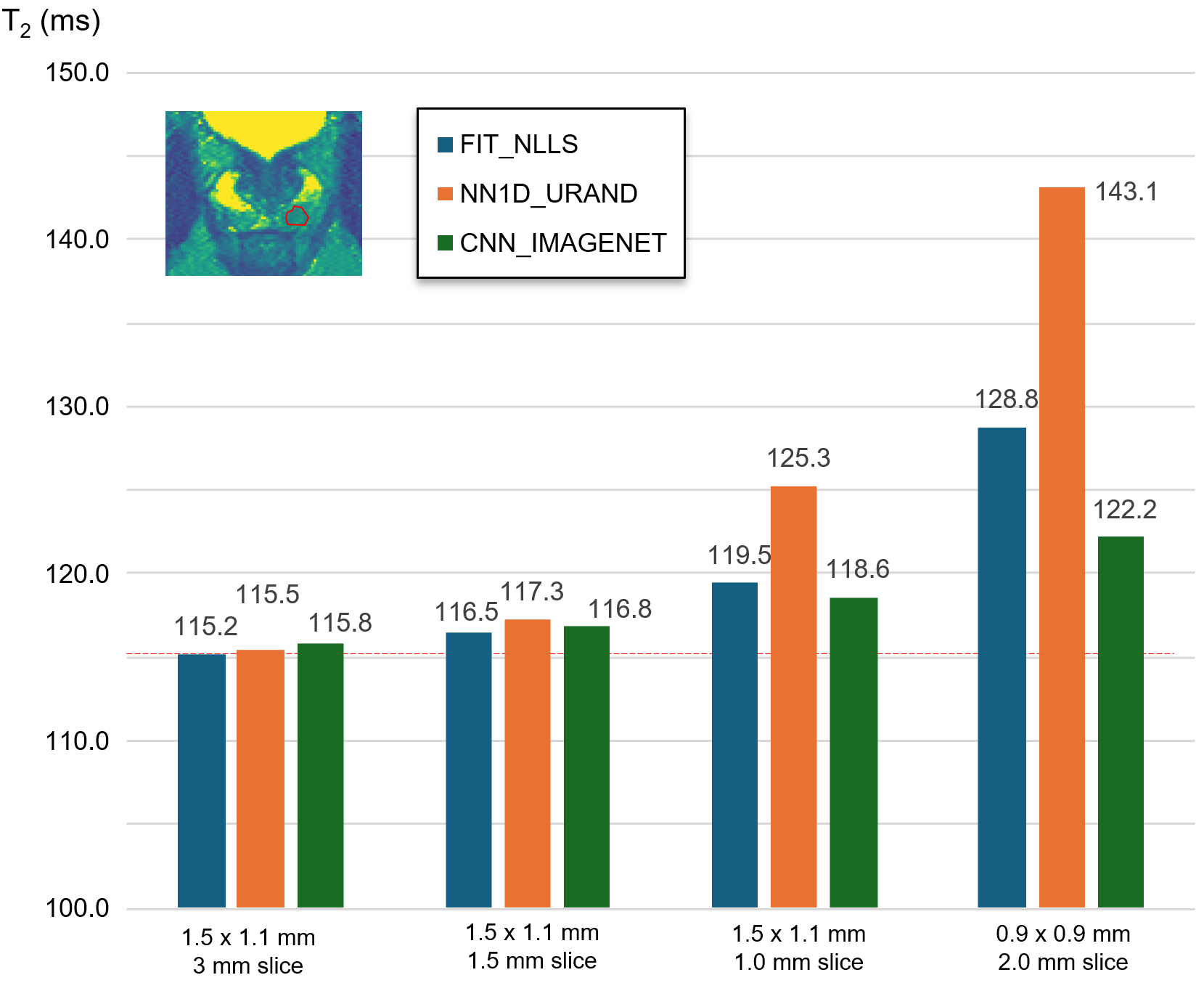


Fig S3 – Effect of decreasing SNR on T_2_ quantification. An ROI (inset) was drawn in a hypointense region of the left peripheral zone for the healthy normal case of Fig S2, and the ROI-mean T_2_ is plotted for each of the three methods and each of the four acquisitions. With decreasing SNR from left to right, the mean T_2_ value in the ROI increases due to noise bias at low SNR. The CNN_IMAGENET acquisition shows lower error (6.1%) than FIT_NLLS (11.8%) and NN1D_URAND (24.2%), relative to the high-SNR FIT_NLLS measurement of 115.2 ms.
